# *In vivo* skin anisotropy dataset from annular suction test

**DOI:** 10.1016/j.dib.2022.107835

**Published:** 2022-01-15

**Authors:** Aflah Elouneg, Audrey Bertin, Quentin Lucot, Vincent Tissot, Emmanuelle Jacquet, Jérôme Chambert, Arnaud Lejeune

**Affiliations:** a Applied Mechanics Department, FEMTO-ST Institute, University Bourgogne Franche-Comté, CNRS (UMR 6174), 24 Rue de L’Épitaphe, Besançon 25000, France; b Biomedical Engineering School at University of Franche-Comté, University Bourgogne Franche-Comté, 23 rue Alain Savary, Besançon 25000, France

**Keywords:** Skin, Anisotropy, Viscoelasticity, Annular suction, *In vivo*, Multi-axial testing, Digital image correlation

## Abstract

To characterize the anisotropic and viscoelastic behaviors of the skin, we conducted an experimental campaign of *in-vivo* suction tests using the CutiScan®CS100 device from Courage and Khazaka electronics. In this data paper, we present the raw acquired data of the tests and their respective treated data. The tests were performed 30 times on the anterior forearm of a 28-year-old Caucasian male at different pressure set-points, ranging from 100 to 500 mbar with an increment of 20 mbar, at ambient temperature in a windowless room. The primary dataset consists of videos recorded by a probe camera associated with the CutiScan® device during the tests. After data treatment with DIC (Digital Image Correlation) technique and based on a homemade Python program, we have obtained secondary data tables and 2D displacement for all mapped grid nodes.

## Specifications Table

**Table:** 

Subject	Material Characterization
Specific subject area	Anisotropic viscoelastic characterization of human skin by digital image correlation technique from multiaxial ring suction test
Type of data	Video Table Image
How data were acquired	Multiaxial ring suction test with CutiScan® probe-camera. Software CS100 Homemade Python program based on PyDIC suite [1]
Data format	Raw Analyzed
Parameters for data collection	The CutiScan® probe was perpendicular to the forearm without any initial press. The observable part of the skin did not contain any hair. The tests were done at ambient temperature and as often as possible at the same time slot.
Description of data collection	Primary raw data are video files (.avi) collected 30 times for 21 different pressure set-points ranging from 100 to 500 mbar with a step size of 20 mbar. Secondary data, consisting of 2D-displacements of all mapped grid nodes, were determined from the primary data through a Python-based program using the digital image correlation technique. they are stored in tables (.csv) and illustrated on images with scaled vectors (.png).
Data source location	Both primary and secondary data have been collected in: Institution: FEMTO-ST Institute, Université Franche-Comté City/Town/Region: Besançon Country: France Latitude and longitude (and GPS coordinates) for collected samples/data: 47°15′04.5″N 5°59′39.6″E (47.251256, 5.994325)
Data accessibility	All data mentioned in this article are available in a public repository. Repository name: *In vivo skin anisotropy dataset for a young man (28 years old) by annular suction* URL to data: https://dmarc.femto-st.fr/fichiers/686/arborescence URL to Python codes: https://dmarc.femto-st.fr/fichiers/687

## Value of the Data

• The data will help analyze human skin's mechanical response when subjected to multi-axial ring suction load. Thus, both anisotropic and viscoelastic phenomena could be explored as the response is temporal and covers all directions, from 0° to 360°.
• Biomechanics researchers can benefit from these data for a complete skin characterization study. Computer scientists could also be interested in using this dataset to improve DIC (Digital Image Correlation) technique.
• This dataset can be combined with data issued from other mechanical tests to identify more independent material parameters.
• The reproducibility of the 30 data series may be either used in the context of Machine Learning training to predict the mechanical behavior efficiently and fast.

## 1 Data Description

There are two folders in the shared public repository, ‘DATA_SUCTION’ and ‘PYTHON_SOURCES.’ The first contains primary and secondary data with a singular ‘README’ text file. This text file provides additional details about the 30-measurement series, such as experiment time slots and data quality, based on a qualitative evaluation of anisotropy distribution stability. While in the second folder, one would find Python codes used to generate secondary data by treating the primary ones.

**Fig. 1:**
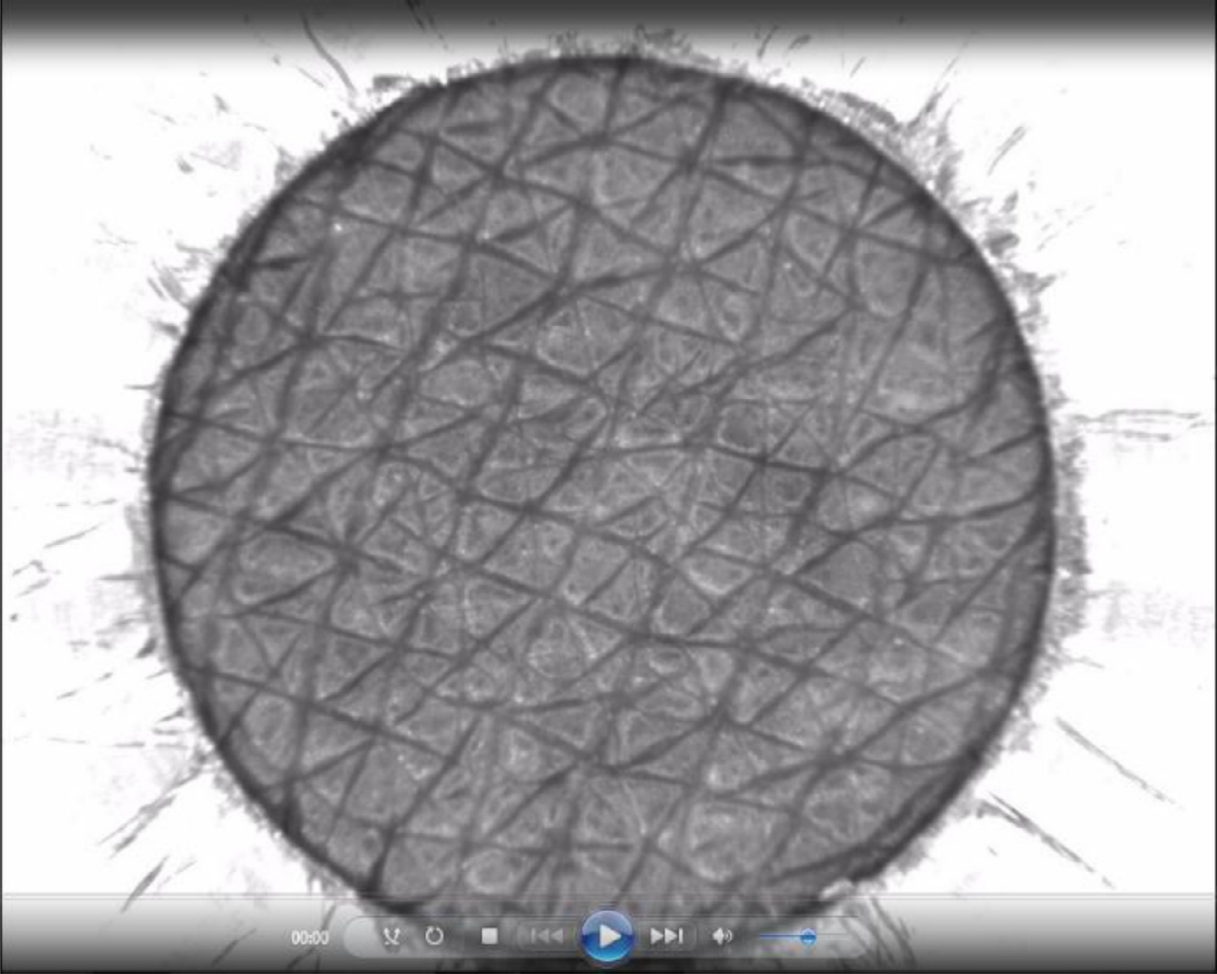
Screenshot of a recorded ring suction test video performed with CutiScan® CS100 probe-camera, taken from the video file ‘2020_12_15\p_300\2020_12_15_300.avi’.

The primary data, video files with ‘.avi’ format, are retrieved for each recorded ring suction test from the commercial software CS100 supplied with the CutiScan®. Fig. 1 represents a snapshot from a video recording, where the dark zone corresponds to the observed skin area. The experiment is duly explained in Section ‘Experimental Design, Materials and Methods’. These video files were subjected to a DIC (Digital Image Correlation) process to track the motions of all Cartesian mapped grid points. Once new positions of the letters are captured for each time-step (frame), we computed their related displacements relative to the initial frame. In addition, but not necessarily, we computed strains and polar coordinates. These secondary data are stored in ‘.csv’ format with a size of 2401 rows and 23 columns (Table 1, Table 2, Table 3) using the scale ‘950 pixel / 5 mm’. The 2401 lines represent the data on the 49 × 49 nodes (rectangular grid with x and y coordinates), while the 23 columns represent respectively:

1. Global index (from 0 to 2400)
2. x-index (from 0 to 48)
3. y-index (from 0 to 48)
4. x-position (pixel)
5. y-position (pixel)
6. x-position (mm)
7. y-position (mm)
8. x-displacement (pixel)
9. y-displacement (pixel)
10. x-displacement (µm)
11. y-displacement (µm)
12. xx-strain
13. yy-strain
14. xy-strain
15. Radial position (pixel)
16. Radial position (mm)
17. Angular position (°)
18. Radial displacement (pixel)
19. Angular displacement (pixel)
20. Radial displacement (µm)
21. Angular displacement (µm)
22. Displacement norm (pixel)
23. Displacement norm (µm)

**Table 1:** Partial view of a ‘.csv’ table with data on every node resulting from DIC process: from column 1 to column 9. The table file path is ‘2020_12_15\p_300\TABLE\frame0041.csv’ (see other columns for the same nodes in Tables 2 and 3).

index	index_x	index_y	pos_x (px)	pos_y (px)	pos_x (mm)	pos_y (mm)	disp_x (px)	disp_y (px)
0	0	0	‒480	480	‒2,526E+00	2,526E+00	‒1,033E+00	9,201E‒01
1	0	1	‒480	460	‒2,526E+00	2,421E+00	‒9,137E‒01	9,255E‒01
2	0	2	‒480	440	‒2,526E+00	2,316E+00	‒7,843E‒01	9,084E‒01
3	0	3	‒480	420	‒2,526E+00	2,211E+00	‒7,259E‒01	9,001E‒01
4	0	4	‒480	400	‒2,526E+00	2,105E+00	‒6,981E‒01	9,049E‒01
5	0	5	‒480	380	‒2,526E+00	2,000E+00	‒7,064E‒01	9,294E‒01
6	0	6	‒480	360	‒2,526E+00	1,895E+00	‒6,502E‒01	9,428E‒01
7	0	7	‒480	340	‒2,526E+00	1,789E+00	‒8,879E‒01	8,674E‒01
8	0	8	‒480	320	‒2,526E+00	1,684E+00	‒7,206E‒01	9,303E‒01
9	0	9	‒480	300	‒2,526E+00	1,579E+00	‒6,804E‒01	9,168E‒01
10	0	10	‒480	280	‒2,526E+00	1,474E+00	‒6,994E‒01	9,614E‒01
11	0	11	‒480	260	‒2,526E+00	1,368E+00	‒6,982E‒01	9,621E‒01
12	0	12	‒480	240	‒2,526E+00	1,263E+00	‒6,642E‒01	9,656E‒01
13	0	13	‒480	220	‒2,526E+00	1,158E+00	‒6,454E‒01	9,501E‒01
14	0	14	‒480	200	‒2,526E+00	1,053E+00	‒6,063E‒01	9,414E‒01
15	0	15	‒480	180	‒2,526E+00	9,474E‒01	‒6,380E‒01	9,766E‒01
16	0	16	‒480	160	‒2,526E+00	8,421E‒01	‒5,911E‒01	9,731E‒01
17	0	17	‒480	140	‒2,526E+00	7,368E‒01	‒5,851E‒01	9,474E‒01
18	0	18	‒480	120	‒2,526E+00	6,316E‒01	‒5,948E‒01	9,487E‒01
19	0	19	‒480	100	‒2,526E+00	5,263E‒01	‒5,995E‒01	9,488E‒01

**Table 2:** Partial view of a ‘.csv’ table with data on every node resulting from DIC process: from column 10 to 17.

disp_x (µm)	disp_y (µm)	strain_xx	strain_yy	strain_xy	r (px)	r (mm)	theta
‒5,436E+00	4,843E+00	0,000E+00	0,000E+00	0,000E+00	6,788E+02	3,573E+00	1,350E+02
‒4,809E+00	4,871E+00	4,306E‒08	‒2,935E‒04	1,468E‒04	6,648E+02	3,499E+00	1,362E+02
‒4,128E+00	4,781E+00	2,016E‒07	‒6,352E‒04	3,177E‒04	6,512E+02	3,427E+00	1,375E+02
‒3,821E+00	4,737E+00	3,816E‒09	‒8,736E‒05	4,368E‒05	6,378E+02	3,357E+00	1,388E+02
‒3,674E+00	4,763E+00	2,679E‒07	7,318E‒04	‒3,658E‒04	6,248E+02	3,289E+00	1,402E+02
‒3,718E+00	4,891E+00	4,497E‒07	9,479E‒04	‒4,737E‒04	6,122E+02	3,222E+00	1,416E+02
‒3,422E+00	4,962E+00	1,198E‒06	‒1,549E‒03	7,752E‒04	6,000E+02	3,158E+00	1,431E+02
‒4,673E+00	4,566E+00	4,892E‒08	‒3,129E‒04	1,565E‒04	5,882E+02	3,096E+00	1,447E+02
‒3,793E+00	4,896E+00	7,610E‒07	1,233E‒03	‒6,161E‒04	5,769E+02	3,036E+00	1,463E+02
‒3,581E+00	4,825E+00	3,022E‒07	7,771E‒04	‒3,884E‒04	5,660E+02	2,979E+00	1,480E+02
‒3,681E+00	5,060E+00	6,422E‒07	1,133E‒03	‒5,660E‒04	5,557E+02	2,925E+00	1,497E+02
‒3,675E+00	5,064E+00	5,623E‒09	1,060E‒04	‒5,302E‒05	5,459E+02	2,873E+00	1,516E+02
‒3,496E+00	5,082E+00	4,495E‒08	‒2,999E‒04	1,500E‒04	5,367E+02	2,825E+00	1,534E+02
‒3,397E+00	5,001E+00	1,830E‒07	‒6,052E‒04	3,027E‒04	5,280E+02	2,779E+00	1,554E+02
‒3,191E+00	4,955E+00	2,183E‒07	6,605E‒04	‒3,301E‒04	5,200E+02	2,737E+00	1,574E+02
‒3,358E+00	5,140E+00	3,136E‒07	7,916E‒04	‒3,957E‒04	5,126E+02	2,698E+00	1,594E+02
‒3,111E+00	5,122E+00	2,649E‒07	‒7,281E‒04	3,642E‒04	5,060E+02	2,663E+00	1,616E+02
‒3,080E+00	4,987E+00	1,867E‒07	‒6,113E‒04	3,057E‒04	5,000E+02	2,632E+00	1,637E+02

**Table 3:** Partial view of a ‘.csv’ table with data on every node resulting from DIC process: from column 18 to 23.

disp_r (px)	disp_theta (px)	disp_r (µm)	disp_theta (µm)	norm_disp (px)	norm_disp (µm)
1,381E+00	7,971E‒02	7,268E+00	4,195E‒01	1,383E+00	7,280E+00
1,300E+00	‒3,598E‒02	6,842E+00	‒1,893E‒01	1,301E+00	6,845E+00
1,192E+00	‒1,396E‒01	6,274E+00	‒7,348E‒01	1,200E+00	6,317E+00
1,139E+00	‒1,994E‒01	5,995E+00	‒1,049E+00	1,156E+00	6,086E+00
1,116E+00	‒2,482E‒01	5,872E+00	‒1,306E+00	1,143E+00	6,015E+00
1,131E+00	‒2,902E‒01	5,951E+00	‒1,527E+00	1,167E+00	6,144E+00
1,086E+00	‒3,642E‒01	5,715E+00	‒1,917E+00	1,145E+00	6,028E+00
1,226E+00	‒1,946E‒01	6,453E+00	‒1,024E+00	1,241E+00	6,533E+00
1,116E+00	‒3,743E‒01	5,872E+00	‒1,970E+00	1,177E+00	6,193E+00
1,063E+00	‒4,168E‒01	5,594E+00	‒2,194E+00	1,142E+00	6,009E+00
1,089E+00	‒4,780E‒01	5,729E+00	‒2,516E+00	1,189E+00	6,257E+00
1,072E+00	‒5,135E‒01	5,643E+00	‒2,702E+00	1,189E+00	6,257E+00
1,026E+00	‒5,666E‒01	5,400E+00	‒2,982E+00	1,172E+00	6,169E+00
9,826E‒01	‒5,948E‒01	5,172E+00	‒3,131E+00	1,149E+00	6,045E+00
9,218E‒01	‒6,358E‒01	4,851E+00	‒3,346E+00	1,120E+00	5,894E+00
9,403E‒01	‒6,904E‒01	4,949E+00	‒3,634E+00	1,166E+00	6,139E+00
8,685E‒01	‒7,363E‒01	4,571E+00	‒3,875E+00	1,139E+00	5,993E+00
8,270E‒01	‒7,457E‒01	4,353E+00	‒3,925E+00	1,114E+00	5,861E+00

**Fig. 2:**
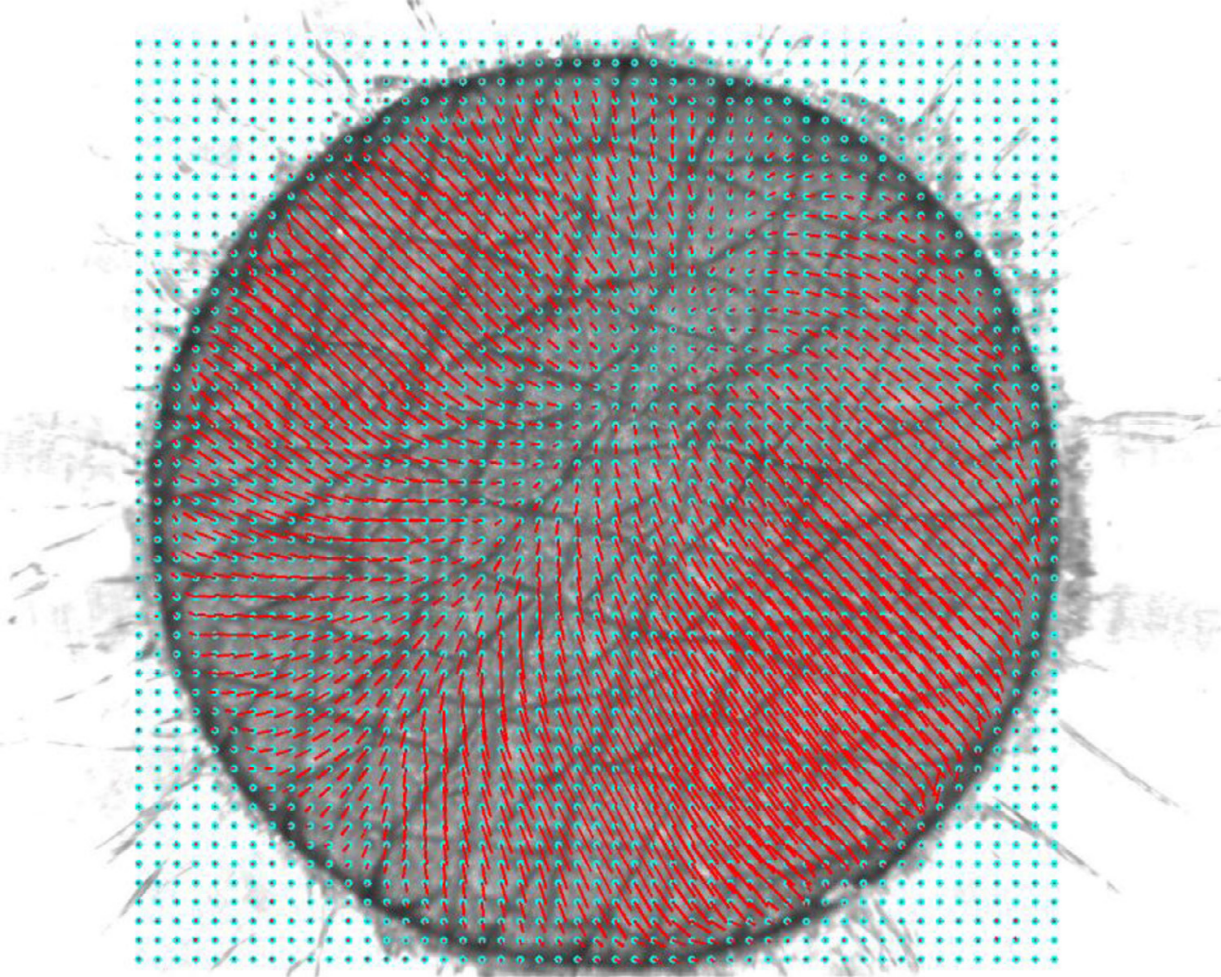
Illustration of displacement field vectors with a scale factor of 5, with an image scale of ‘950 pixel / 5 mm’. The blue points represent the grid nodes with a gap of 20 pixels and a correlation window size of 72 pixels. The image file path is ‘2020_12_15\p_300\DISP_FIG\frame0041_disp.png’.

Finally, the content of columns 4,5,8, and 9 have been used to map 2D-displacement vectors on nodes with a scale factor of 5, using the reference image as background (Fig. 2). Displacement vectors are plotted in red on every node.

‘PYTHON_SOURCES’ repository contains Python programs based on PyDIC suite methods [1], which were used to generate CSV tables from AVI videos. The code sources are detailed in Section ‘Experimental Design, Materials and Methods’.

## 2 Experimental Design, Materials and Methods

**Fig. 3:**
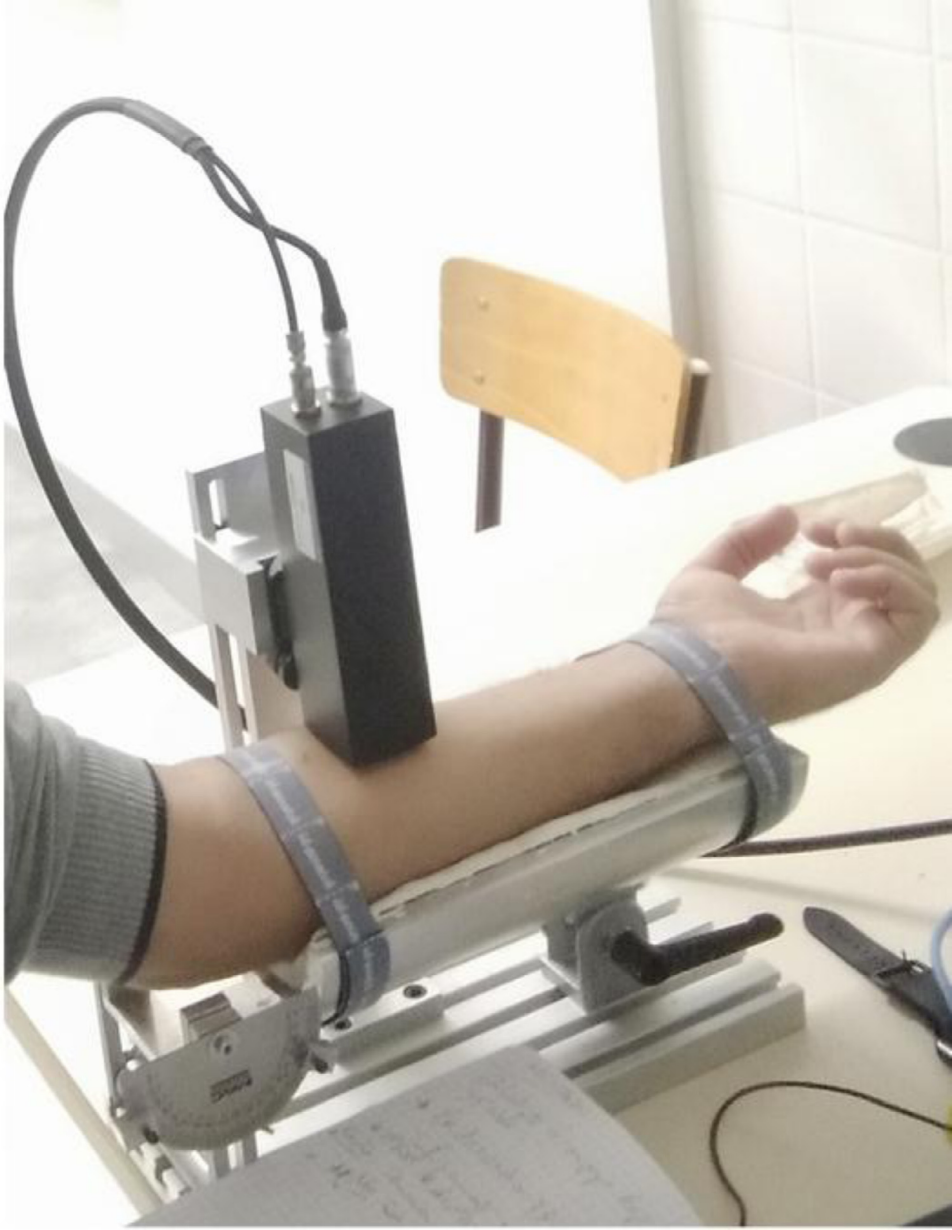
Application of Cutiscan® on human forearm anterior.

The primary measurement videos have been captured for 30 ring suction test series performed on the anterior forearm of a 28-years-old Caucasian male using CutiScan® probe-camera [2], as often as possible at the same time slot. The forearm was positioned perpendicular to the probe (Fig. 3). Laiacona et al. [3] used a similar homemade device with a 30 mm-diameter measurement zone.

**Fig. 4:**
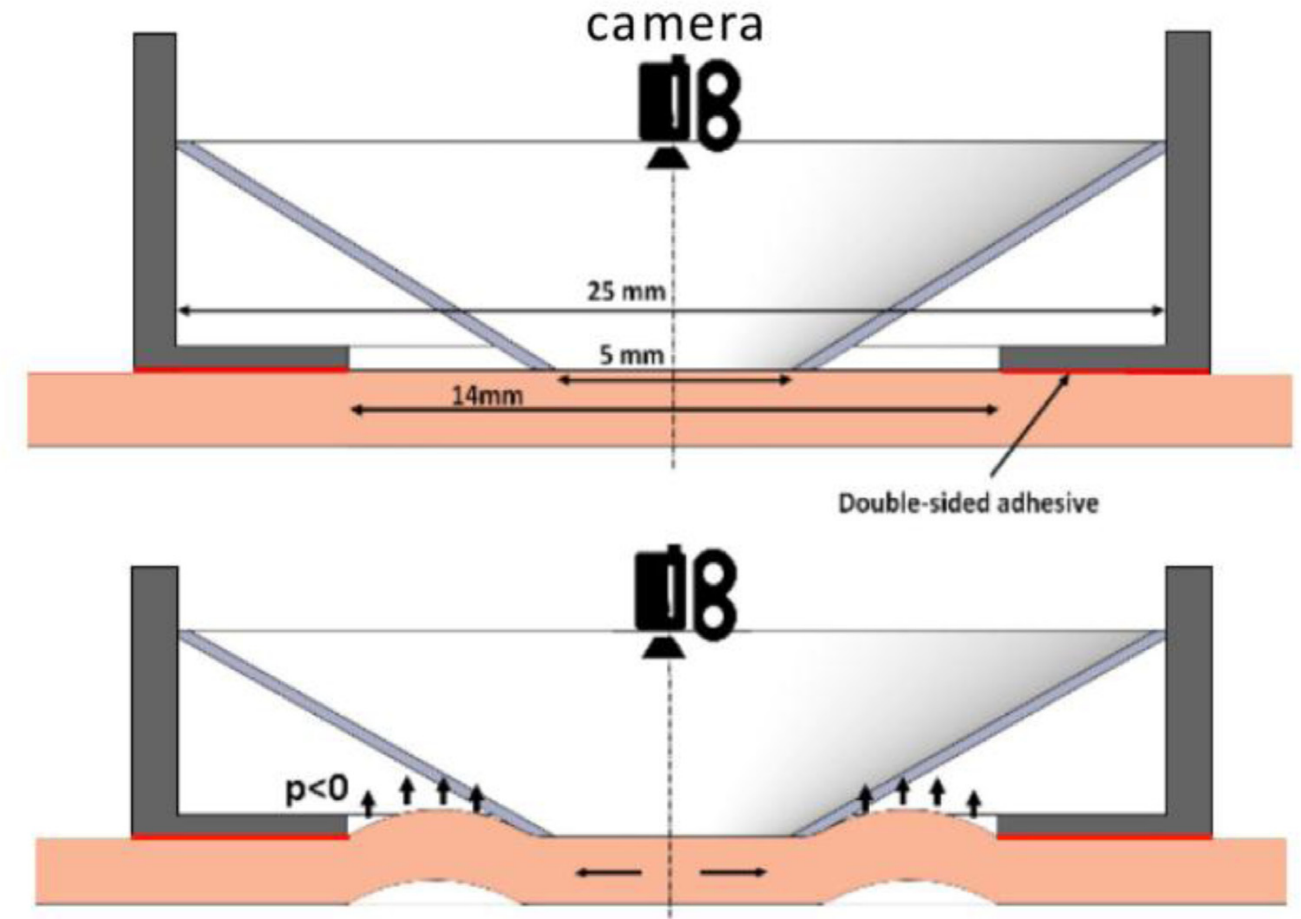
Cutiscan® probe-camera cross-section [4].

Fig. 4 illustrates the working principle of the ring suction test. Negative pressure was applied within an annular section, around the measured central zone. The inner and outer diameters of the ring are, respectively, 5 mm and 14 mm. The central zone is subjected to in-plane multi-axial extension. The pink medium represents the skin layer, and black arrows display the direction of the ring area displacement.

**Fig. 5:**
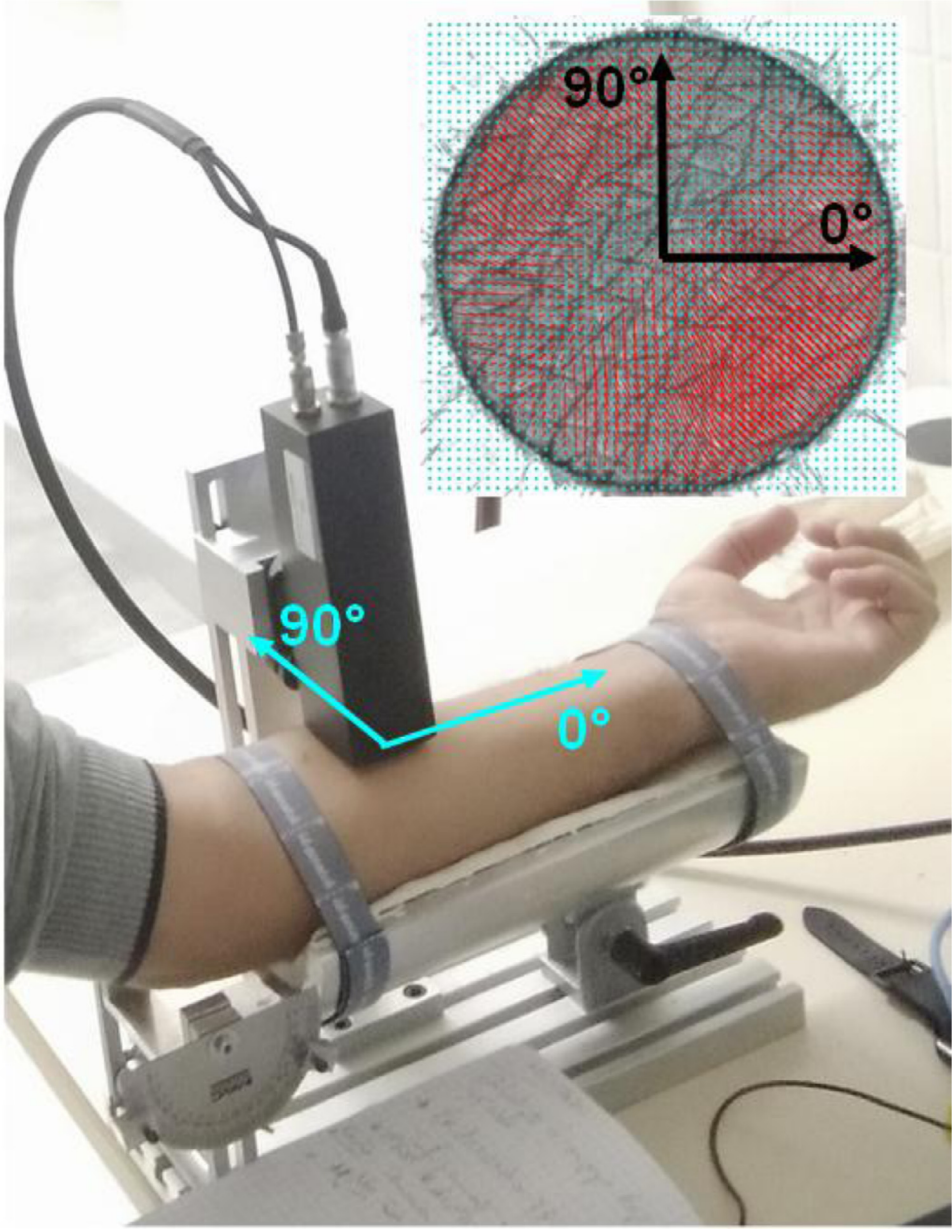
Orientation axis of probe and observation zone.

A double-sided adhesive ring tape was placed around the measurement area to ensure a good seal between the probe and the skin (Fig. 4). The tape is stuck to the probe oriented along the zero-angle of the camera (Fig. 5).

**Fig. 6:**
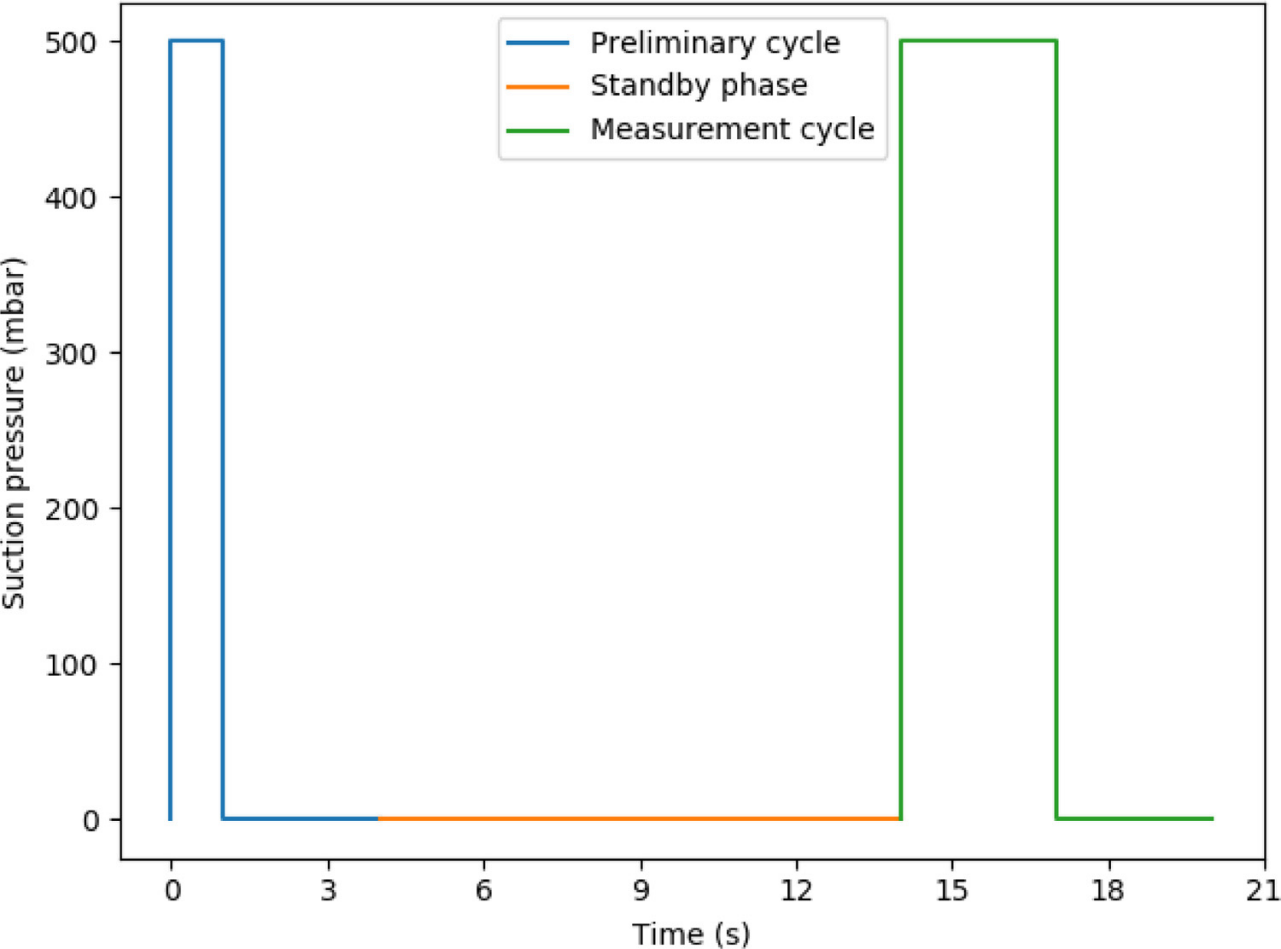
Pressures set-points of the two loading cycles.

Load application phases were carried as follows (Fig. 6):

- Primary cycle: apply the set aspiration pressure instantaneously, hold it for 1 s, and then release it for 3 s.
- Standby phase: wait for the skin to recover its properties for 10 s
- Measurement cycle: apply instantaneously the set aspiration pressure and hold it for 3 s, then release it for 3 s.

This routine was repeated for 21 negative pressure set-points varying from 100 to 500 mbar with a step of 20 mbar. A delay of 2 min between each measurement is required to allow the skin to recover its viscoelastic properties. Hence, a daily data series lasts around 1 h.

As the role of the primary cycle is stabilizing the UV lightening inside the probe, only the measurement cycle is recorded by µEye® camera (Fig. 6). The 6 s video file is then retrieved from CS100, a ‘black box’ software provided with the device. An alternative DIC program based on the PyDIC suite [1] and compatible with data formats has been implemented by our team. PyDIC suite use Lucas-Kanade method optical flow algorithms [5] implemented in OpenCV (Computer Vision) Python-library [6]. The relative correlation parameters: correlation grid resolution and correlation window size are, respectively, 20 pixels and 72 pixels. Those parameters, fixed on the basis of error optimization study, can be easily modified in the algorithm.

Video files, considered in this paper as primary data, are processed by this program to create tables in the form of ‘.csv’ (Table 1). The displacement fields are illustrated in ‘.png’ images (Fig. 2). To generate those secondary data, the user needs to execute the Python (version >= 3.6) code source whose path is ‘PYTHON_SOURCE/multi_test.py’:

*>> python3 multi_test.py*

**Fig. 7:**
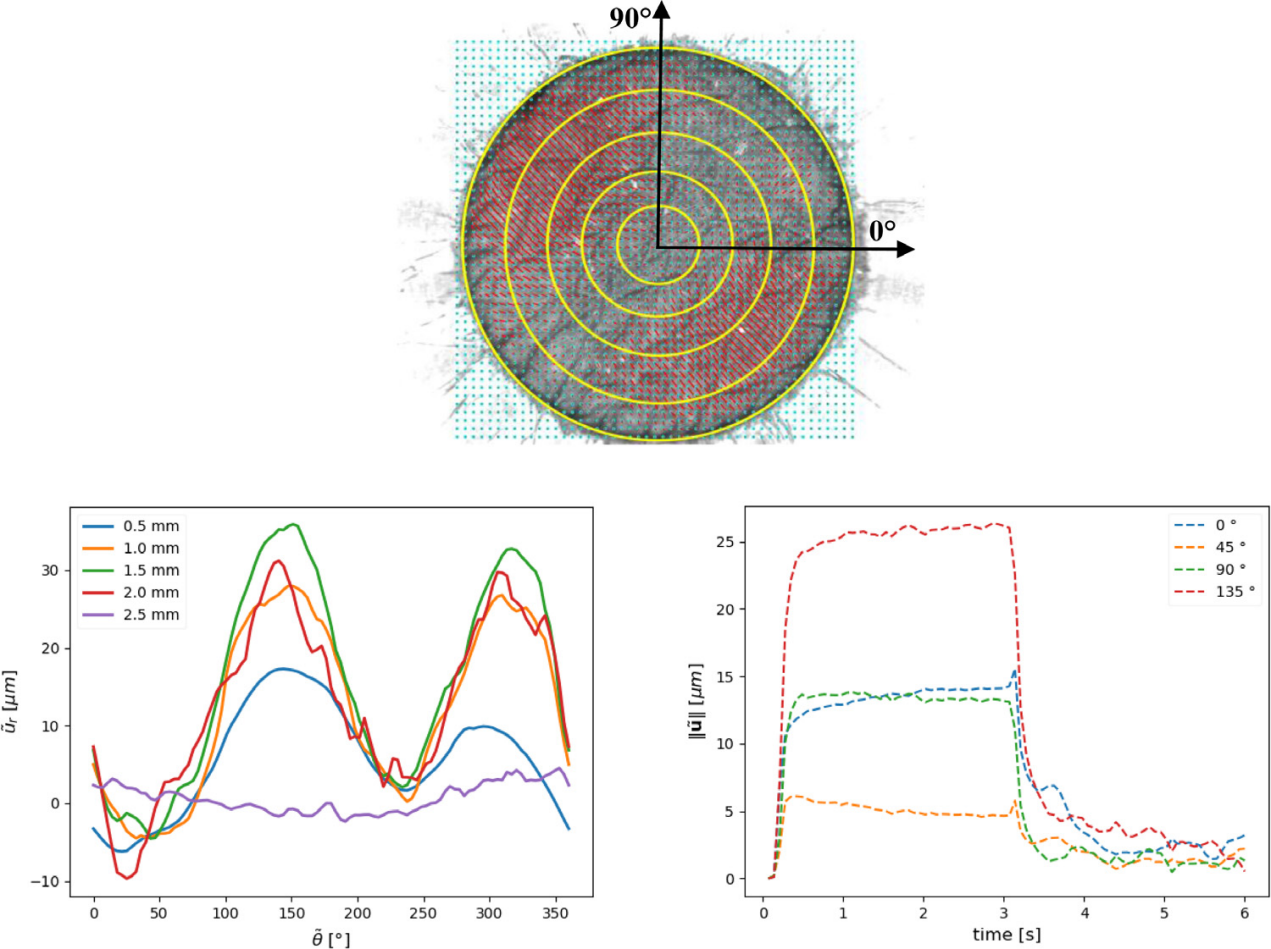
A preview of derived graphs from temporal-spatial data analysis.

Several plots can be derived from the secondary dataset. As an example in Fig. 7 for a given series and pressure set-point (files path: ‘2020_12_14\p_300\TABLES), the radial component of the displacement ${\tilde{u}}_{r}$ evaluated by linear interpolation on the five yellow circles with radius r={0.5;1;1.5;2;2.5} mm has been plotted with respect to the angle $\tilde{\theta }$. Also, the evolution of the displacement norm $∥\tilde{a}∥$ over time has been represented for different angles $\tilde{\theta }=\left\{0^{\circ },\;45^{\circ },\;90^{\circ },\;135^{\circ }\right\}$ along the circle r=1 mm. The Python codes used to generate the graphs are available in https://dmarc.femto-st.fr/fichiers/691.

## Ethics Statement

The described work has been carried out in accordance with The Code of Ethics of the World Medical Association (Declaration of Helsinki) for experiments involving humans. All data have been collected with the patient's consent, and the patient's privacy is fully preserved. Recently, we have obtained approval from the Committee for the Protection of Persons (CPP) to conduct a similar experimental campaign on 30 subjects.

## CRediT authorship contribution statement

**Aflah Elouneg:** Formal analysis, Writing – review & editing, Visualization, Investigation, Software. **Audrey Bertin:** Writing – original draft, Investigation. **Quentin Lucot:** . **Vincent Tissot:** . **Emmanuelle Jacquet:** Conceptualization, Supervision. **Jérôme Chambert:** Conceptualization, Supervision. **Arnaud Lejeune:** Data curation, Conceptualization, Supervision.

## Declaration of Competing Interest

The authors declare that they have no known competing financial interests or personal relationships which have or could be perceived to have influenced the work reported in this article.
